# Aging transition by random errors

**DOI:** 10.1038/srep42715

**Published:** 2017-02-15

**Authors:** Zhongkui Sun, Ning Ma, Wei Xu

**Affiliations:** 1 Department of Applied Mathematics, Northwestern Polytechnical University, Xi’an 710129, China

## Abstract

In this paper, the effects of random errors on the oscillating behaviors have been studied theoretically and numerically in a prototypical coupled nonlinear oscillator. Two kinds of noises have been employed respectively to represent the measurement errors accompanied with the parameter specifying the distance from a Hopf bifurcation in the Stuart-Landau model. It has been demonstrated that when the random errors are uniform random noise, the change of the noise intensity can effectively increase the robustness of the system. While the random errors are normal random noise, the increasing of variance can also enhance the robustness of the system under certain conditions that the probability of aging transition occurs reaches a certain threshold. The opposite conclusion is obtained when the probability is less than the threshold. These findings provide an alternative candidate to control the critical value of aging transition in coupled oscillator system, which is composed of the active oscillators and inactive oscillators in practice.

Oscillatory behavior is essential for proper functioning of natural and/or artificial system(s), hence is universal and inevitable in real-world. Consequently, modeling coupled oscillators provide one an alternative way to detect the oscillation pattern of physical and biological processes, and help to understand the dynamic complexity of the real nonlinear subjects in diverse fields ranging from physics, biology and chemistry to engineering^1^,^2^,^3^,^4^. Therefore, coupled nonlinear oscillators has attracted tremendous attention during the past decade, and several typical motions have been reported, as examples, chaos and synchronization^5^,^6^,^7^,^8^,^9^,^10^, amplitude death or restoration^11^,^12^,^13^, and aging transition^14^.

Aging transition is an oscillating behavior that coupled oscillator systems, composed of active and inactive oscillators, lose its macroscopic activity measured by a global order parameter of amplitude, *Z*, as the increase of the ratio of inactive oscillators, *p*, until it totally vanished at a certain critical value *p*_*c*_, which can be utilized to characterize the robustness of the dynamical system^15^,^16^,^17^. Aging transition is a common phenomenon in real-world, which is first proposed and investigated by Daido and Nakanishi^14^ in 2004 to study the robustness of the activity of the coupled oscillator system so as to oppose the dynamic aging caused by various damages or deterioration. Hereafter, the behavior of aging transition has received wide attention^18^,^19^,^20^,^21^,^22^,^23^. Daido *et al*. discussed the behavior of aging transition in systems of different topology, including globally and diffusively coupled oscillators with the emphasis on the desynchronization of active oscillators^18^, coupled oscillators that parameter values are not uniform^19^, a large ring of coupled oscillators^20^, and coupled heterogeneous oscillators^16^, which make the application of aging transition spread more widely. In 2014, Huang and his co-authors^21^ promoted aging transition to networked oscillators, which is helpful to design effective strategies to enhance or destroy the dynamical robustness in real-world. Further, Thakur *et al*.^22^ discussed the effects of time delay on the aging transition in coupled oscillators, and found that time delay might facilitate the aging transition by reducing the critical coupling strength of amplitude death in the system.

Commonly, it is difficult for researchers or engineers to determine accurately a parameter in natural or artificial systems, because of the limitation of measuring tools, calculating approaches and/or environmental factors. Consequently, the measurement error is unavoidable in a real system, and has been the subject of much research in mathematics, physics and engineering. Many efforts have been devoted to estimating or eliminating errors, and some wonderful results have been reported in the past several decades^24^,^25^. However, the effects of errors on the dynamical behaviors, e.g., vibration and stability, in real systems have received few attentions, consequently, which have remained elusive to date. Inspiredly, this paper focuses on measurement errors of parameters and aims at getting an insight into the effects of measurement errors on the oscillating behavior in coupled oscillators, which, to the authors’ best knowledge, has not been reported before.

Without loss of generality, the measurement error is represented by random noise (or random error equally) according to error theory in current study, and the oscillator in every single note is a Stuart-Landau model, in which random errors have been assumed to being with the parameter that specifies the distance from a Hopf bifurcation. Two kinds of typical noises have been employed in current paper to describe the random errors accompanied with the bifurcation parameter. It is expected to find out how the random errors impact the oscillating motion of the nonlinear oscillator array.

## Results

### Coupled Stuart-Landau oscillators

The globally coupled Stuart-Landau equation is described as follows^14^,^26^,^27^,^28^,^29^,^30^:


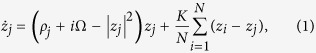


for *j* *=* 1, …, *N*, where the overdot means differentiation with respect to time *t, z*_*j*_ is the complex amplitude of the *j*th oscillator, Ω is the natural frequency, *K* > 0 is the coupling strength, and *ρ*_*j*_ is the parameter specifying the distance from a Hopf bifurcation. According to ref. 14, oscillators are active if *ρ*_*j*_ = *a* > 0, and inactive if *ρ*_*j*_ = −*b* < 0. Aging occurs when the active oscillators switch to the inactive ones. Therefore, one can divide all of the oscillators into two groups: one group contains all active elements: *j* ∈ {1, …, *N*(1 − *p*)} ≡ *S*_*a*_ and another group includes the inactive ones: *j* ∈ {*N*(1 − *p*) + {1, …, *N*} ≡ *S*_*i*_, where *p* is the ratio of inactive elements. Setting *z*_*j*_ = *A* for all active elements and *z*_*j*_ = *I* for all inactive elements, then the original model can be simplified^14^ into


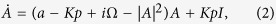



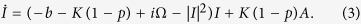


The eigenvalues can be obtained from the simplified equations (2) and (3). So aging transition will emerge when all the real parts of the eigenvalues are not positive.

Setting Δ = (*b* − *a* + *K*^2^) + 4*a(b* + *K*) − 4*K(a* + *b)p*, then,

if Δ ≥ 0, the real parts of the eigenvalues are


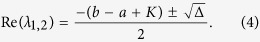


Letting Re(*λ*_1,2_) ≤ 0, one can get


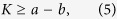


and


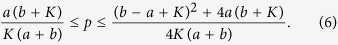


If Δ < 0, one obtains


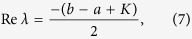


and


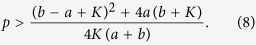


**Figure 1:**
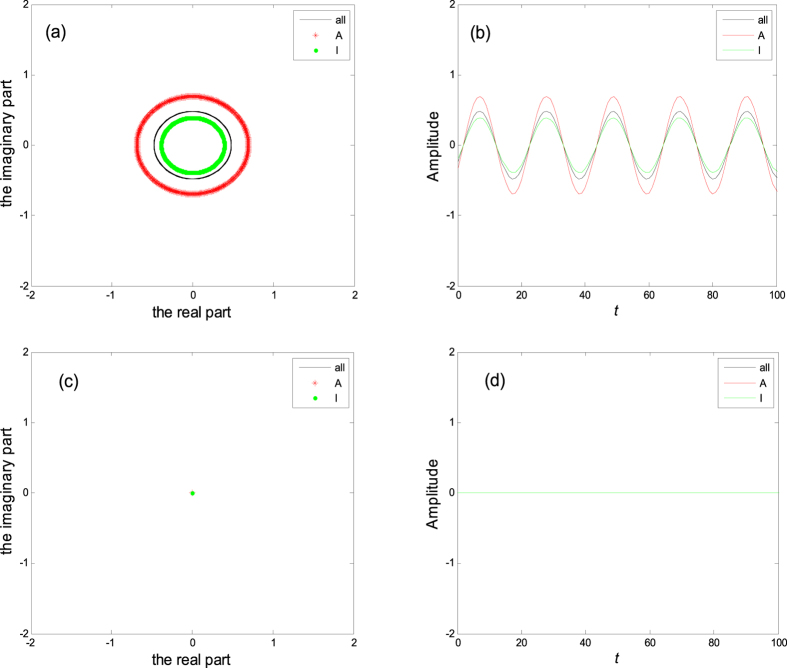
All the figures show trajectories of *z*_*j*_for *K* = 5. (**a**) and (**c**) are in the complex plane with the abscissa and ordinate meaning the real and imaginary part, where *p* = 0.4 in (**a**), *p* = 0.9 in (**c**). (**b**) and (**d**) are time history with the same *p* as (**a**) and (**c**), where ***A*** represent active elements, ***I*** represent inactive elements, ***all*** represent both active and inactive elements.

Since *p* is the ratio of inactive elements, Equations (6) and (8) can be rearranged in the following form^14^,


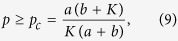


where *K* ≥ *a*. This implies that if the ratio of inactive elements *p* is more than *p*_*c*_, the system will fall into the trivial fixed point *z*_1_ = L = *z*_*N*_ = 0, which we call quiescent phase, otherwise the system will oscillate in perfect synchronization, as shown in Fig. 1. Hence, *p*_*c*_ indi_*c*_ates the critical value of aging transition.

**Figure 2:**
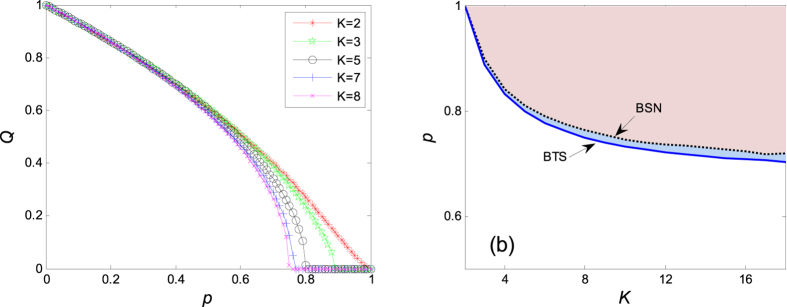
Aging in the coupled Stuart-Landau equations, where *N* = 1000, *a* = 2, *b* = 1, Ω = 3. *Q* = |*Z(p*)|/|*Z*(0)|. (**a**) Variations of transition points in coupled Stuart-Landau systems under different coupling strength. (**b**) The ratio of inactive oscillators *p* is plotted against the coupling strength *K*, where BSN is the boundary of the numerical simulation, while BTS is the boundary of the theoretical solution which is obtained from Equation (9). The filled areas in the top right corner are the aging transition areas. Aging transition is considered to occur if the order parameter *Q* < 0.005. In the upper right area of Fig. 2(b), the system is in the quenching state losing global activity, while in the bottom left area, it’s oscillating to some extent.

An order parameter |Z| is introduced for characterizing the behavior of aging transition^14^, where 
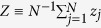
. Its normalized format is defined as *Q*=|*Z(p*)|/|*Z*(0)|, which is plotted in the left panel of Fig. 2. One reads clearly that the normalized order parameter *Q* decreases monotonously with the increase of *p*, until *Q* = 0 at *p*_*c*_, announcing the appearance of aging transition. Figure 2(b) shows the theoretical solution (9) and the numerical one, through which a good agreement can be found between the numerical solution and the theoretical one.

### Uniform random errors

#### The distance parameter of active oscillators

In this section, we discuss the aging behaviors in the globally coupled Stuart-Landau oscillators when the distance parameter of the active oscillators has been affected by uniform random errors. Namely, the distance parameter of *a* changes from *a* to *a* + *ξ*, with *ξ* ~ *U*[0, *r*]. The simplified equations (2) and (3) are recast as


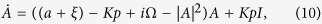



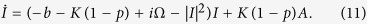


By the Lyapunov stability theory, one can derive the conditions of Re*λ* ≤ 0,


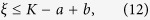


and


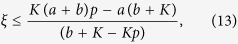


where Δ = *ξ*^2^ + 2(*a* + *b* + *K* − 2*Kp)ξ* + [(*a* + *b* + *K*)^2^ − 4*Kp(a* + *b*)].

Since *ξ* is a uniform random noise instead of a constant, its value is not fixed or constant, and can not been determined in advance. As a result, one cannot estimate the critical value of aging transition from in equations (12) and (13) directly because of the dependence on *ξ*. Accordingly, we try to derive the conditions of aging transition in the coupled Stuart-Landau oscillators when the distance parameters are disturbed by random errors in the framework of probability theory.

Provided that the coupled oscillators access into the behavior of aging with the probability no less than *α (α* denotes the confidence level), then one can derive the conditions of aging transition based on probability skills,


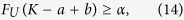


and


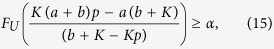


where *F*_*U*_(g) is the uniform distribution function with


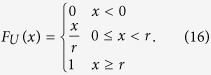


So the condition of aging transition is


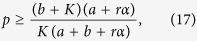


where *K* ≥ *ra* + *a*, and 
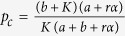
 is the critical value of aging transition with the probability no less than *α*. It shows the dependence of *p*_*c*_ on the confidence level *α* and other parameters.

**Figure 3:**
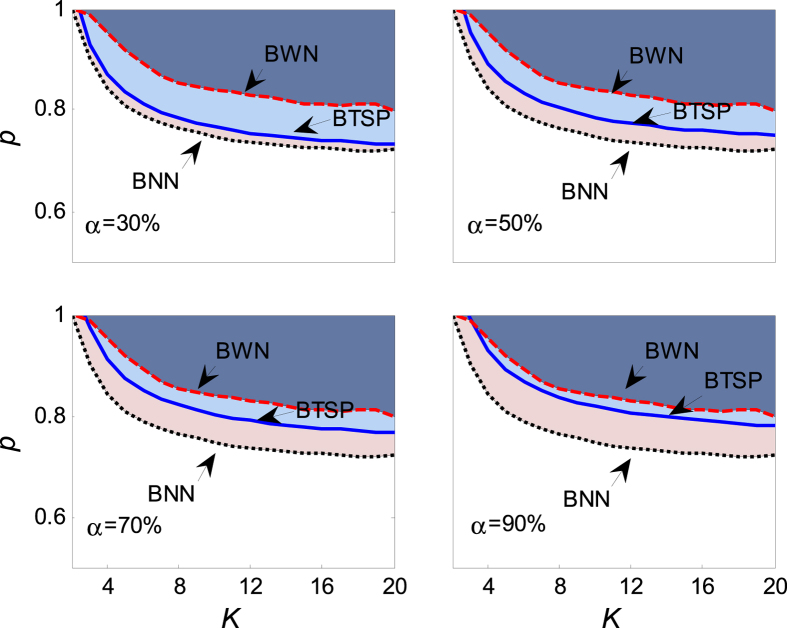
The dependence of the critical value of aging transition *p*_*c*_ on the coupling strength *K* in the presence of uniform random error *ξ*. Therein, *N* = 1000, *r* = 1, *a* = 2, *b* = 1 and Ω = 3. In the panels, BNN (the black dotted line) is the boundary of *p*_*c*_ when the coupled oscillators (1) contain no random errors, which is calculated numerically from Equations (2) and (3); BTSP (the blue solid line) is the boundary of the theoretical solution, which is determined and solved by Equation (17); BWN (the red dashed line) is the boundary of *p*_*c*_ when containing random errors, which is solved numerically from Equations (10) and (11). The shadowing area in the panels denotes the field of aging transition. All the numerical boundaries are obtained by averaging over 100 realizations of noise from equations (10) and (11) by means of the fourth order Rung-Kutta method, and the transition is confirmed when Q < 0.005, hereafter.

**Figure 4:**
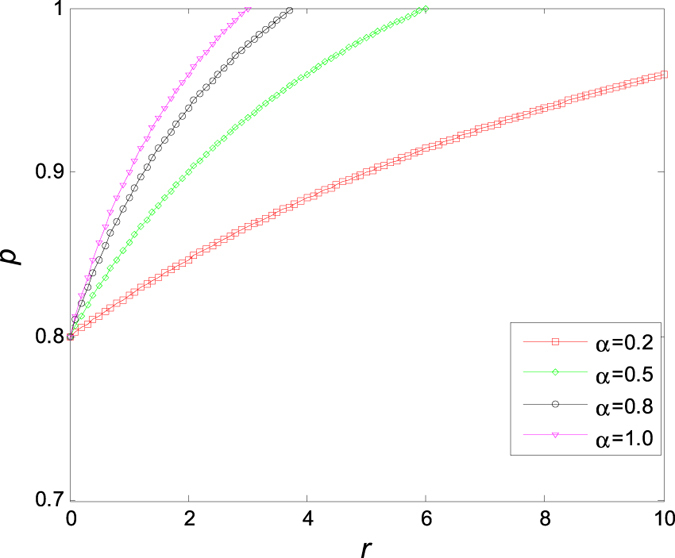
The critical value *p*_*c*_ is plotted against the right boundary of the uniform random error for different *α*, where the coupling strength is *K* = 5.

**Figure 5:**
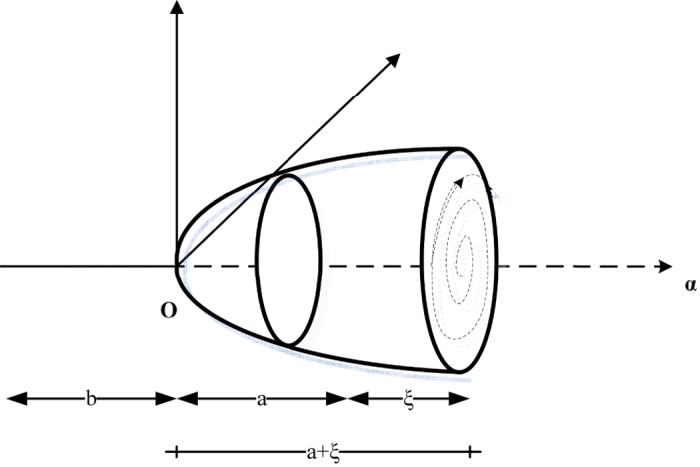
Hopf bifurcation diagram.

It is important to note from Fig. 3 that the quiescent phase *p* > *p*_*c*_ recedes as the confidence level *α* grows. When the confidence level increase to 1, namely *α* = 1, the behavior of aging transition will occur in this region with probability 1. One can read that the theoretical results will get closer to the numerical simulation with the bigger *α*. The dependence of the aging transition boundary *p* = *p*_*c*_(*r, α*) is plotted in Fig. 4 to verify the result obtained in Fig. 3. It shows that: (i) Above the boundary curve *p*_*c*_, the oscillators will be attracted to the trivial fixed point; and (ii) below the boundary, the oscillators will remain dynamic. The critical *p*_*c*_ is a monotonically increasing function of *r* for each *α* as shown in Fig. 4, which reflects the fact that the uniform random error *ξ* ~ *U*[0, *r*] enlarges the distance to the Hopf bifurcation, hence leads to the increase of the amplitude of active oscillators, just as shown in Fig. 5. Therefore, it’s more difficult for the coupled oscillators to achieve globally synchronized oscillation. Namely, the uniform random errors enhance the robustness of the globally coupled Stuart-Landau oscillators when it disturbs the distance parameter of the active oscillators.

#### Distance parameter of all the oscillators

In this section, we study the effects of the uniform random error *ξ* ~ *U*[0, *r*] on the distance parameters of both the active and the inactive oscillators.

In the presence of the uniform random errors, the simplified equations will be rewritten as


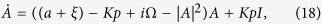



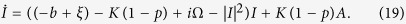


Analogizing to the previous section, the conditions of Re*λ* ≤ 0 are


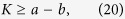


and


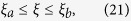


where *ξ*_*a*_, *ξ*_*b*_ are the roots of


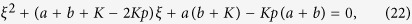


satisfying *ξ*_*a*_ < *ξ*_*b*_. Assuming that the probability of aging transition is not less than *α*, that is,


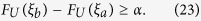


**Figure 6:**
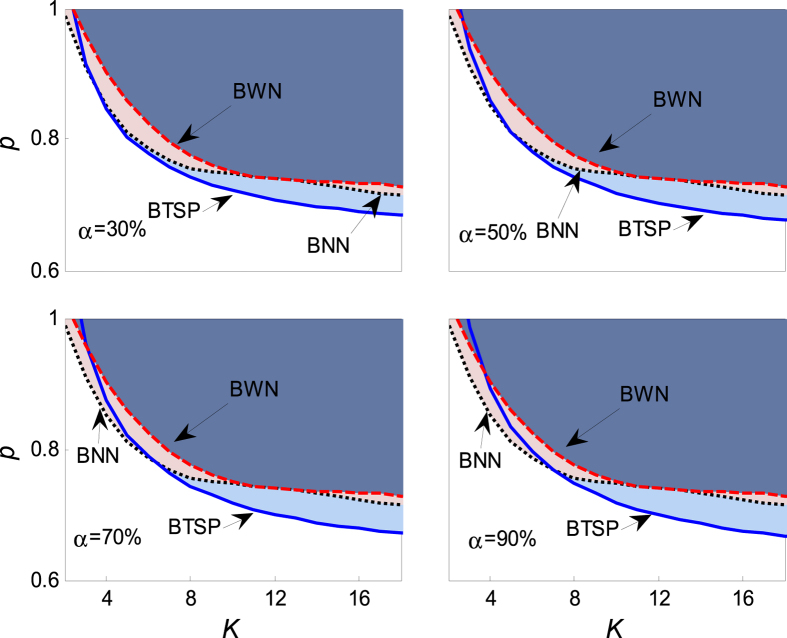
The dependence of the critical value of aging transition *p*_*c*_ on the coupling strength *K* in the presence of uniform random error *ξ*. The parameters are fixed the same as those in Fig. 3. In the panels, BNN is calculated numerically from Equations (2) and (3), BTSP is solved by Equation (24), and BWN is determined numerically from Equations (18) and (19). Every numerical point is obtained by averaging over 100 realizations of noise from equations (18) and (19).

**Figure 7:**
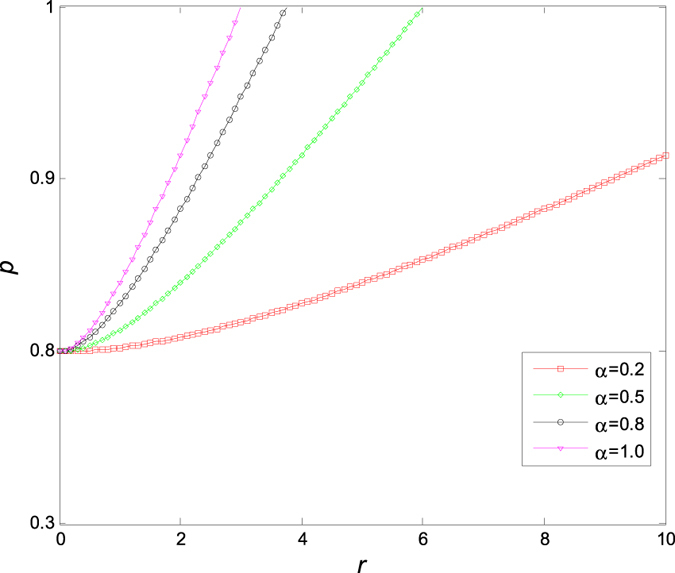
The critical value *p*_*c*_ is plotted against the right boundary of the uniform random error for different *α*, where the coupling strength is *K* = 5.

Then, one can get the critical value of aging transition based on probability theory,


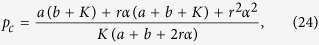


which means that aging transition occurs with the probability no less than *α* when *p* ≥ *p*_*c*_. The dependence of the critical value *p*_*c*_ on the coupling strength *K* is plotted in Fig. 6 for different *α*, from which one observes that the critical value decreases as the increase of the coupling strength. And the numerical result agrees well with the theoretical solution (24). Comparing with the critical value of aging transition without noise errors, the quiescent phase with the uniform random error is less than that without the uniform random error, which implies that the existence of the uniform errors enhances the robustness of the coupled oscillators. Figure 7 displays the dependence of the critical value *p*_*c*_ on the noise intensity *r*. It is found that *p*_*c*_ is an increasing function of *r*, which demonstrates that a stronger noise will help the coupled oscillators maintaining oscillating, hence enhance the robustness.

### Normal random errors

#### Distance parameter of active oscillators

In this section, the behavior of aging transition is discussed in globally coupled Stuart-Landau equations when the bifurcation parameters are affected by normal random errors. At first, we consider the case where the active oscillators are affected by normal random errors. The simplified model is


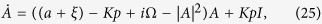



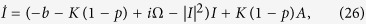


with *ξ* ~ N(*μ, σ*^2^).

The real parts of the eigenvalues are


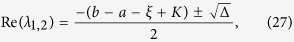


where Δ = *ξ*^2^ + 2(*a* + *b* + 2*Kp)ξ* + [(*a* + *b* + *K*)^2^ − 4*Kp(a* + *b*)] ≥ 0. Setting Re*λ* ≤ 0, one can obtain.


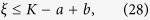


and


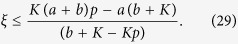


Evidently, when


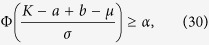


and


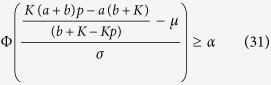


the behavior of aging transition will take place with the probability no less than *α*. Therein, Φ(g) is the standard normal distribution function. By means of probability skills, we obtain the condition of aging transition with probability no less than *α*,


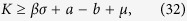


and


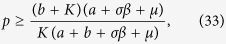


where Φ(*β*) = *α*, and 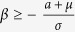 or 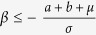.

**Figure 8:**
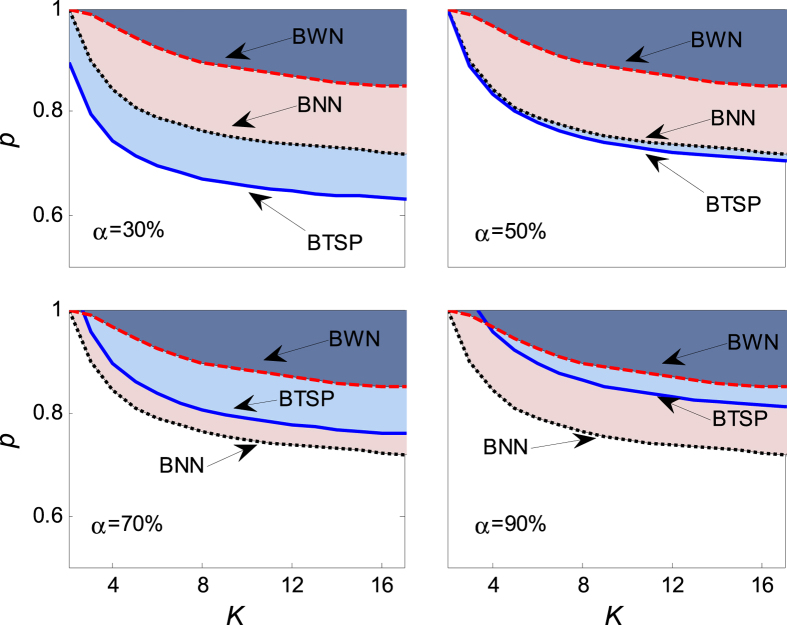
The dependence of the critical value of aging transition *p*_*c*_ on the coupling strength *K* in the presence of normal random error *ξ*. The values of parameters are chosen as *N* = 1000, *μ* = 0, *σ* = 1, *a* = 2, *b* = 1 and Ω = 3. In the panels, BNN is calculated numerically from Equations (2) and (3), BTSP is solved by Equation (33), and BWN is determined numerically from Equations (25) and (26). The numerical boundaries are obtained by averaging over 100 realizations of noise from equations (25) and (26).

Figure 8 exhibits the critical value of aging transition for different *a*. It is found that the critical value *p*_*c*_ decreases with the increase of coupling strength *K*, but it is an increasing function of the probability *α*. When the probability is not less than *α* = 30%, the theoretical quiescent phase is much bigger than the numerical one. Increasing the probability *α*, the theoretical result becomes indistinctive with the numerical value, as the example, one can check the last panel with *α* = 90%. On the other hand, one can read from Fig. 8 that the quiescent phase with noise is much smaller than that without noise, therefore, which verifies that the normal random errors can enhance evidently the robustness of the coupled oscillators.

**Figure 9:**
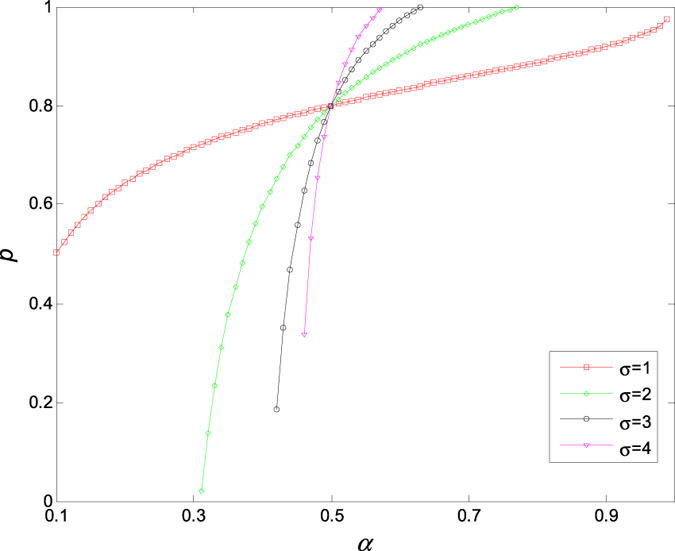
The critical value *p* of aging transition changes with the probability *α* and the variance *σ* of random errors *ξ*. Therein, *μ* = 0 and *K* = 5.

In Fig. 9, we show the dependence of critical value *p*_*c*_ on the probability *α* for different variance *σ* of random errors *ξ*, from which one can read immediately that *p*_*c*_ increases with the probability *α*, and there exists a critical point (*α* *=* 50%, *p*_*c*_ = 0.8). Evidently, *p*_*c*_ decreases with the increase of *σ* when *α<*50%,, and increases with the increase of *σ* when *α* *>* 50%, which implies that the variance of the random error plays an important role in maintaining oscillation of the coupled Stuart-Landau models, particularly, when the confidence level is higher than 50%.

It is worth mentioning that it can happen that *a* + *ξ* is negative when the distance parameter is affected by the normal random noise, in which case all oscillators of the network are intrinsically “inactive”. One can derive that P(*a* + *ξ* < 0) = 0.0228 by means of the probability density function of normal distribution. Namely, there is a 2.28% chance of *a*+*ξ*<0. While it happens, all the oscillators will turn into the inactive ones, which is too trivial to further discuss.

#### Distance parameter of all the oscillators

Here we study, as before, such a case that all the oscillators are affected by the same type of random errors. The simplified model is


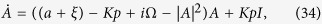



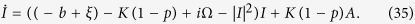


with *ξ* ~ N(*μ, σ*^2^). The conditions of Re*λ* ≤ 0 are


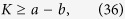


and


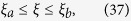


where *ξ*_*a*_ and *ξ*_*b*_ are the roots of characteristic equation *ξ*^2^ + (*a* + *b* + *K* − 2*Kp)ξ* + *a(b* + *K*) − *Kp(a* + *b*) ≤ 0. Thus the condition that aging transition occurs with the probability no less than *α* can be derived as


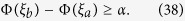


**Figure 10:**
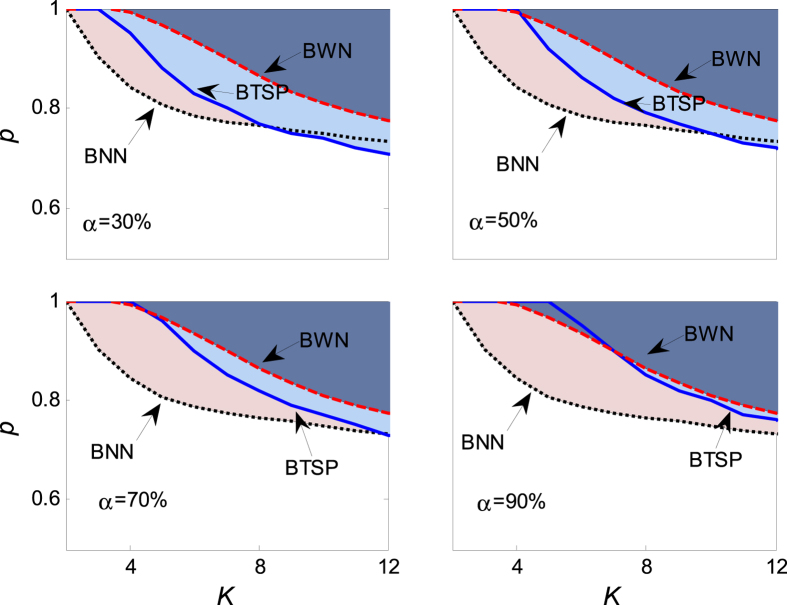
The dependence of the critical value of aging transition *p*_*c*_ on the coupling strength *K* in the presence of normal random error *ξ*, for *μ* = 1, *σ* = 1, *N* = 1000, *a* = 2, *b* = 1, and Ω = 3. In the panels, BNN is calculated numerically from Equations (2) and (3), BTSP is solved by Equation (38), and BWN is determined numerically from Equations (34) and (35). The numerical boundaries are obtained by averaging over 100 realizations of noise from equations (34) and (35).

Figure 10 shows the critical value changed with the coupling strength and the probability of aging transition when the distance parameters of both the active and the inactive oscillators are impacted by the standard normal random errors. Analogous phenomena and concludes can be summarized as the ones of Fig. 8.

## Discussion

To summarize, the effects of the random errors on the aging transition have been investigated in this paper. Two typical noises, say uniform random noise and normal random noise, are employed to affect the bifurcation parameters *ρ*, respectively. Due to the uncertainty of the random errors, the critical value of aging transition *p*_*c*_ is not fixed or constant anymore, who dependents on the random errors and therefore possesses randomicity. Hence, we studied the aging oscillations in the coupled Stuart-Landau oscillators and built the representations of the critical value of aging transition *p*_*c*_ in the framework of probability theory, based on which different parameters of the random errors have been discussed to analyze the influences on the aging behaviors. It has been found that: (i) In the case of uniform random noise, the existence of random errors enhance the robustness of the system with the increase of the errors’ level; (ii) In the case of normal random noise, the random errors enhance the robustness of the oscillator like the uniform random noise, but the variance of the normal random error may affect the robustness in different ways: if *α* > 50%, the critical value of aging transition *p*_*c*_ increases with the increase of the variance if *α* > 50%, but decreases with the variance if *α* < 50%. Evidently, the random errors play the important roles in inducing or suppressing aging oscillations in coupled oscillator systems, which, in some level, states the crucial influents of measurement errors of parameters on oscillating dynamics in a network of coupled oscillators.

## Additional Information

**How to cite this article**: Sun, Z. *et al*. Aging transition by random errors. *Sci. Rep.* **7**, 42715; doi: 10.1038/srep42715 (2017).

**Publisher's note:** Springer Nature remains neutral with regard to jurisdictional claims in published maps and institutional affiliations.
